# A New Optical Method for Suppressing Radial Magnetic Error in a Depolarized Interference Fiber Optic Gyroscope

**DOI:** 10.1038/s41598-018-20487-x

**Published:** 2018-01-31

**Authors:** Yanru Zhou, Yuxiang Zhao, Dengwei Zhang, Xiaowu Shu, Shuangliang Che

**Affiliations:** 1 State Key Laboratory of Modern Optical Instrumentation, Zhejiang University, Hangzhou, 310027 Zhejiang China; 2 Beijing System Design Institute of Mechanical-Electrical Engineering, Beijing, 100854 China

## Abstract

Based on the theory of the radial magnetic error (RME) in depolarized interference fiber optic gyroscopes (D-IFOGs) under magnetic field, a new optical method is proposed to decrease the RME by adding a suppressing section fiber (SSF) in D-IFOGs. A related theoretical model is established, and the solutions of the parameters of the SSF are obtained with numerical calculations. Then the results of the suppressed RME are simulated. An experimental system is set up to verify the theory and simulation, and the experimental results prove that the RME can be suppressed effectively with a SSF added in the D-IFOG. The magnitude of the RME can be reduced to one-tenth of the original.

## Introduction

**Figure 1:**
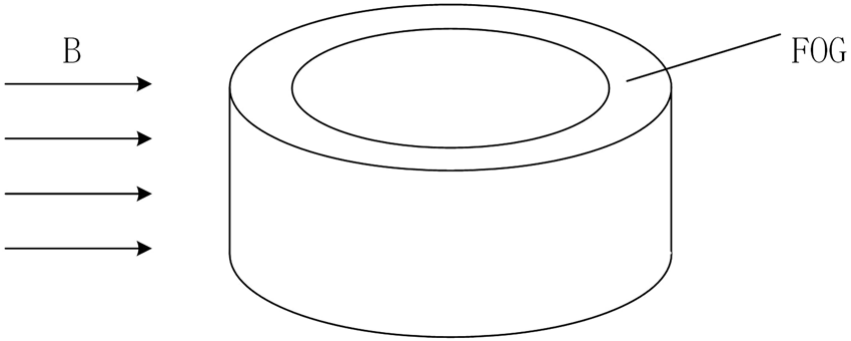
Radial magnetic field.

The main kinds of optic gyroscope include interference fiber optic gyroscope (IFOG), resonator fiber optic gyroscope (RFOG) and ring laser gyroscope (RLG)^1^. In IFOG, the light propagates in fiber. When the fiber coil is put in a magnetic field, there is Faraday effect in silica fiber. And the radial magnetic error results from Faraday effect^2^. As for RFOG, the resonator features with a length of only tens meters, even several micrometers. Sometimes, the resonator is made by photonic crystal fiber instead of traditional solid-core fiber^3^. Considering the short length of resonator and the use of photonic crystal fiber, the Faraday effect can be effectively weakened. In RLG, since the light propagates in air^4,5^, the Faraday effect is much weaker than in silica fiber. Above all, the magnetic error is small and can be ignored in RFOG and RLG considering their special structures. But in IFOG, it is necessary to study and suppress the magnetic error. Depolarized Interference Fiber Optic Gyroscope (D-IFOG) is one kind of the important Interferometric Fiber Optic Gyroscope (IFOG). When there is radial magnetic field vertical to the axis of the fiber coil, the output of the fiber optic gyroscope (FOG) will be changed. Here we define the output error of the FOG (caused by the unit radial magnetic field) as the radial magnetic error (RME). Assuming that Ω_0_ is the output of FOG when there is no magnetic field, Ω_1_ is the output of FOG when there is radial magnetic field and B is the magnitude of the radial magnetic field. Then the RME can be expressed as (Ω_1_ − Ω_0_)/*B*. And the radial magnetic field is shown in Fig. 1. The radial magnetic error (RME) in D-IFOG derives from the Faraday Effect^2^. RME is related to several parameters including the intensity and orientation of the magnetic field, the length of the fiber and the 45° error of the depolarizer. RME is also significantly influenced by the linear birefringence, the magnitude and the distribution of the fiber twist on the coil^6–9^. In practice, the inevitable environmental magnetic field induces RME changing along with the orientation of the magnetic fields. It affects the measurement accuracy of the angular velocity severely. Therefore, RME is one of the remarkable problems in medium-to-high precision IFOGs in reality.

Currently, there are three main methods to decrease the magnetic error, including magnetic shielding, software compensation, fiber coil compensation. Magnetic shielding is widely adopted in engineering^10–12^, but it will obviously increase the weight and volume of IFOG^13^. As for software compensation, a small magnetometer in IFOG measures the magnetic field and the magnetic error can be compensated with software. Nevertheless, this method is focused on axial magnetic field only^14,15^. In fiber coil compensation, several particular fiber coils are joined in IFOG to compensate the RME^16,17^. However it suffers from some weaknesses, such as the difficulty of operation, the low consistency of the compensation and the degradation of the temperature characteristics in IFOG.

Based on previous studies, we propose an optical method to suppress the RME in this paper. Between the depolarizer and fiber coil, a section of single mode fiber (SMF) is inserted as a suppressing section fiber (SSF). By adjusting key parameters of the SSF, the RME of D-IFOG can be decreased effectively. With the theory of the RME in D-IFOG, a theoretical model of the optical method for suppressing the RME is established. The solutions of the parameters of the SSF are obtained with numerical calculations. An experimental system is set up to verify the theory and simulation. The results turn out that the magnitude of the RME can be reduced to one-tenth of the original.

## The theoretical model of the optical method for suppressing RME

The RME is closely related to the linear birefringence, the distribution of the twist on the fiber coil, the intensity and orientation of the magnetic field^6^. In a certain fiber coil, there must be linear birefringence and twist, which will produce dramatic RME in D-IFOG under magnetic field. The reduction of RME can be realized by adding a section of SSF. By adjusting the twist and linear birefringence of the added fiber, the two beams along the clockwise (CW) orientation and the counter clockwise (CCW) orientation in the fiber produce approximately equivalent phase variety when there is only radial magnetic field and no rotation. In other words, the magnetic sensitivity of the D-IFOG will be greatly decreased. Then the destination of reducing the RME can be realized.

**Figure 2:**
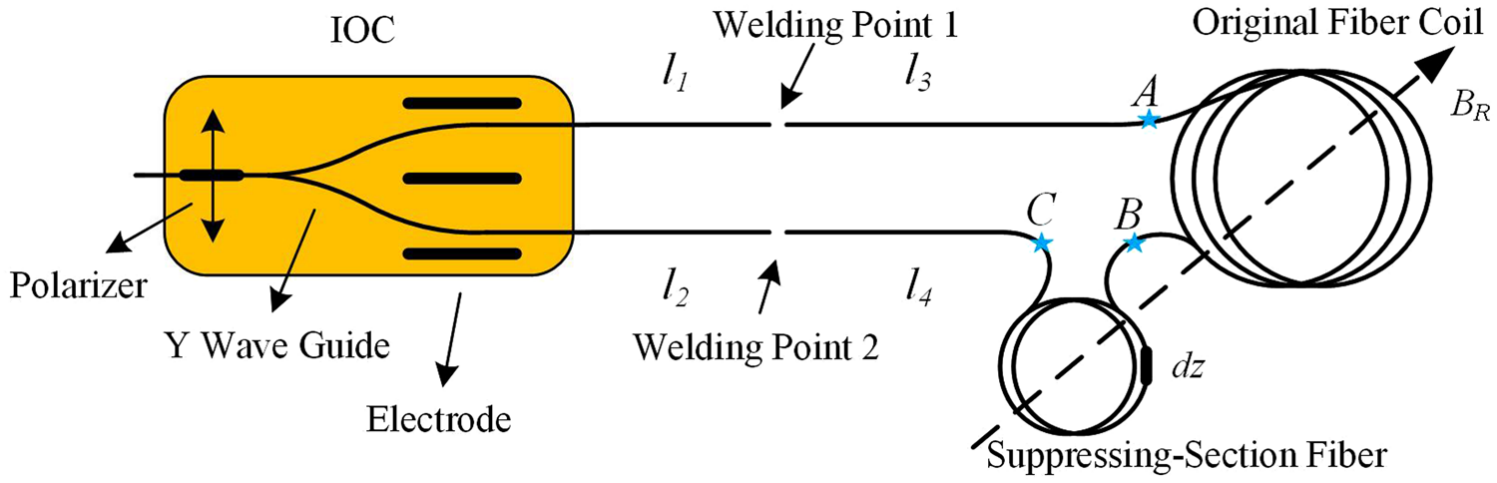
Optical scheme for RME suppression in D-IFOG.

Figure 2 is the optical scheme of the RME suppression in D-IFOG. The light source and detector are omitted. As shown in Fig. 1, the integrated optical component (IOC) consists of polarizer, Y wave guide and electrodes used for phase modulation and feedback. The IOC can be equivalent to an ideal polarizer. *l*_1_~*l*_4_ are four sections of polarization maintaining fiber (PMF) with the linear birefringence of Δ*β*_*b*_. *l*_1_ and *l*_3_ constitute a depolarizer, as well as *l*_2_ and *l*_4_. The 1# and 2# are two welding points with designed angle of 45°, while the actual angles are *θ*_1_ and *θ*_2_ respectively. The 45° errors of 1# and 2# are Δ*θ*_1_ and Δ*θ*_2_. *B*_*R*_ represents the radial magnetic field. The original fiber coil is between point A and B. The SSF is located between point B and C. The length, linear birefringence and twist of the SSF will be determined after calculating and simulating.

According to the ref.^18^, the transmission matrixes of the light in fiber are calculated based on an infinitesimal method. Assuming that the length of the SSF is *L*_*s*_ and the SSF is divided into *m*_*s*_ pieces, the length of the i-th piece is d*z* = *L*_*s*_/*m*_*s*_. When the beam along CW orientation goes through the SSF, the sequence numbers of the little pieces are 0, 1, …, i, …, *m*_*s*_ − 2, *m*_*s*_ − 1, orderly. Then the transmission matrix of CW light for the whole SSF is,

1 $$[\begin{array}{cc}{A}_{s} & {B}_{s}\\ -{{B}_{s}}^{\ast } & {{A}_{s}}^{\ast }\end{array}]=\prod _{i={m}_{s}-1}^{0}{u}_{cs,i}$$

where^19,20^,

2 $${u}_{cs,i}=[\begin{array}{cc}\cos ({\eta }_{cs,i}dz)-j\frac{{\rm{\Delta }}{\beta }_{s}(i)}{2{\eta }_{cs,i}}\,\sin ({\eta }_{cs,i}dz) & -\frac{[{\phi }_{s}(i)+\zeta (i)]}{{\eta }_{cs,i}}\,\sin ({\eta }_{cs,i}dz)\\ \frac{[{\phi }_{s}(i)+\zeta (i)]}{{\eta }_{cs,i}}\,\sin ({\eta }_{cs,i}dz) & \cos ({\eta }_{cs,i}dz)+j\frac{{\rm{\Delta }}{\beta }_{s}(i)}{2{\eta }_{cs,i}}\,\sin ({\eta }_{cs,i}dz)\end{array}]$$

3 $${\eta }_{cs,i}=\sqrt{{[{\rm{\Delta }}{\beta }_{s}(i)/2]}^{2}+{[{\phi }_{s}(i)+\zeta (i)]}^{2}}$$

Equation (2) is the transmission matrix of the CW light in the i-th piece. Δ*β*_*s*_(*i*) is the linear birefringence of the SSF. *φ*_*s*_(*i*) and *ζ*(*i*) is the circular birefringence of the SSF induced by twist and Faraday Effect respectively. *ζ*(*i*) = V*B(*i*), where *V* is the Verdet constant of the material, *B*(*i*) is the intensity of the magnetic field in the i-th piece. When the CW light passes through the whole nonreciprocal light path, the transmission matrix is given by

4 $$\begin{array}{rcl}{U}_{cs} & = & [\begin{array}{cc}1 & 0\\ 0 & 0\end{array}]T[{\rm{\Delta }}{\beta }_{b}{l}_{2}]C[{\theta }_{2}]T[{\rm{\Delta }}{\beta }_{b}{l}_{4}][\begin{array}{cc}{A}_{s} & {B}_{s}\\ -{{B}_{s}}^{\ast } & {{A}_{s}}^{\ast }\end{array}]\\  &  & \times [\begin{array}{cc}A & B\\ -{B}^{\ast } & {A}^{\ast }\end{array}]\,T[{\rm{\Delta }}{\beta }_{b}{l}_{3}]C[{\theta }_{1}]T[{\rm{\Delta }}{\beta }_{b}{l}_{1}][\begin{array}{cc}1 & 0\\ 0 & 0\end{array}]\\  & = & [\begin{array}{cc}{{\rm{\Gamma }}}_{cs} & 0\\ 0 & 0\end{array}]\end{array}.$$

Where $${\rm{T}}[{\rm{\Delta }}{\beta }_{b}l]=[\begin{array}{cc}\exp (-j{\rm{\Delta }}{\beta }_{b}l/2) & 0\\ 0 & \exp (j{\rm{\Delta }}{\beta }_{b}l/2)\end{array}]$$ is the transmission matrix in PMF, and $${\rm{C}}[\theta ]=[\begin{array}{cc}cos\theta  & -sin\theta \\ sin\theta  & cos\theta \end{array}]$$ is the rotation matrix in welding point. $$[\begin{array}{cc}A & B\\ {B}^{\ast } & {A}^{\ast }\end{array}]$$ is the transmission matrix of the CW light in the original fiber coil. The expression of $$[\begin{array}{cc}A & B\\ {B}^{\ast } & {A}^{\ast }\end{array}]$$ is similar to $$[\begin{array}{cc}{A}_{s} & {B}_{s}\\ {B}_{s}^{\ast } & {A}_{s}^{\ast }\end{array}]$$, except that *u*_*cs*_, Δ*β*_*s*_, *φ*_*s*_, *η*_*cs*_ are substituted by *u*_*c*_, Δβ, φ, *η*_*c*_ of the original fiber coil.

Similarly, the transmission matrix of the beam along CCW orientation for the whole SSF is

5 $$[\begin{array}{cc}{C}_{s} & {D}_{s}\\ -{{D}_{s}}^{\ast } & {{C}_{s}}^{\ast }\end{array}]=\prod _{i=0}^{{m}_{s}-1}{u}_{ccs,i}$$

Where

6 $${u}_{ccs,i}=[\begin{array}{c}\cos ({\eta }_{ccs,i}dz)-j\frac{{\rm{\Delta }}{\beta }_{s}(i)}{2{\eta }_{ccs,i}}\,\sin ({\eta }_{ccs,i}dz)\\ \frac{[{\phi }_{s}(i)-\zeta (i)]}{{\eta }_{ccs,i}}\,\sin ({\eta }_{ccs,i}dz)\end{array}\begin{array}{c}-\frac{[{\phi }_{s}(i)-\zeta (i)]}{{\eta }_{ccs,i}}\,\sin ({\eta }_{ccs,i}dz)\\ \cos ({\eta }_{ccs,i}dz)+j\frac{{\rm{\Delta }}{\beta }_{s}(i)}{2{\eta }_{ccs,i}}\,\sin ({\eta }_{ccs,i}dz)\end{array}]$$

7 $${\eta }_{ccs,i}=\sqrt{{[{\rm{\Delta }}{\beta }_{s}(i)/2]}^{2}+{[{\phi }_{s}(i)-\zeta (i)]}^{2}}$$

Equation (6) is the transmission matrix of the CCW light in the i-th piece. When the CCW light passes through the whole nonreciprocal light path, the transmission matrix is given by

8 $$\begin{array}{rcl}{U}_{ccs} & = & [\begin{array}{cc}1 & 0\\ 0 & 0\end{array}]T[{\rm{\Delta }}{\beta }_{b}{l}_{1}]C[{\theta }_{1}]T[{\rm{\Delta }}{\beta }_{b}{l}_{3}][\begin{array}{cc}C & D\\ -{D}^{\ast } & {C}^{\ast }\end{array}]\\  &  & \times [\begin{array}{cc}{C}_{s} & {D}_{s}\\ -{{D}_{s}}^{\ast } & {{C}_{s}}^{\ast }\end{array}]T[{\rm{\Delta }}{\beta }_{b}{l}_{4}]C[{\theta }_{2}]T[{\rm{\Delta }}{\beta }_{b}{l}_{2}][\begin{array}{cc}1 & 0\\ 0 & 0\end{array}]\\  & = & [\begin{array}{cc}{{\rm{\Gamma }}}_{ccs} & 0\\ 0 & 0\end{array}]\end{array}$$

Where $$[\begin{array}{cc}C & D\\ {D}^{\ast } & {C}^{\ast }\end{array}]$$ is the transmission matrix of the CCW light in the original fiber coil. The expression of $$[\begin{array}{cc}C & D\\ {D}^{\ast } & {C}^{\ast }\end{array}]$$ is similar to $$[\begin{array}{cc}{C}_{s} & {D}_{s}\\ {D}_{s}^{\ast } & {C}_{s}^{\ast }\end{array}]$$, except that *u*_*ccs*_, Δ*β*_*s*_, *φ*_*s*_, *η*_*ccs*_ are substituted by *u*_*cc*_, Δβ, φ, *η*_*cc*_ of the original fiber coil.

There is a $\frac{{\rm{\pi }}}{2}$ phase modulation in IFOG. Therefore the interference intensity of the light along the CW and CCW orientation can be shown as

9 $${I}_{s}(\nu )={\rm{Re}}[({{\rm{\Gamma }}}_{cs}+{{\rm{\Gamma }}}_{ccs}\cdot \exp (-{\rm{j}}\frac{\pi }{2}))\cdot {({{\rm{\Gamma }}}_{cs}+{{\rm{\Gamma }}}_{ccs}\cdot \exp (-{\rm{j}}\frac{\pi }{2}))}^{\ast }]=2+\text{2sin}({\rm{\Delta }}{\varphi }_{{\rm{Bs}}})$$

Where Δ*ϕ*_*Bs*_ is the phase difference of CW light and CCW light after adding SSF.

Assuming that

10 $$\begin{array}{rcl}[\begin{array}{cc}{A}_{s} & {B}_{s}\\ -{{B}_{s}}^{\ast } & {{A}_{s}}^{\ast }\end{array}]\,[\begin{array}{cc}A & B\\ -{B}^{\ast } & {A}^{\ast }\end{array}] & =\,[\begin{array}{cc}{A}_{s}A-{B}_{s}{B}^{\ast } & {A}_{s}B+{A}^{\ast }{B}_{s}\\ -{{A}_{s}}^{\ast }{B}^{\ast }-A{{B}_{s}}^{\ast } & {{A}_{s}}^{\ast }{A}^{\ast }-{{B}_{s}}^{\ast }B\end{array}] & =\,[\begin{array}{cc}A\text{'} & B\text{'}\\ -B{\text{'}}^{\ast } & A{\text{'}}^{\ast }\end{array}]\\ \,[\begin{array}{cc}C & D\\ -{D}^{\ast } & {C}^{\ast }\end{array}]\,[\begin{array}{cc}{C}_{s} & {D}_{s}\\ -{{D}_{s}}^{\ast } & {{C}_{s}}^{\ast }\end{array}] & =\,[\begin{array}{cc}C{C}_{s}-D{{D}_{s}}^{\ast } & C{D}_{s}+{{C}_{s}}^{\ast }D\\ -{C}^{\ast }{{D}_{s}}^{\ast }-{C}_{s}{D}^{\ast } & {C}^{\ast }{{C}_{s}}^{\ast }-{D}^{\ast }{D}_{s}\end{array}] & =\,[\begin{array}{cc}C\text{'} & D\text{'}\\ -D{\text{'}}^{\ast } & C{\text{'}}^{\ast }\end{array}]\end{array}$$

the expression of complex amplitude of every item in matrix (10) is written as

11 $$\begin{array}{c}{A}^{\text{'}}={a}^{\text{'}}\exp ({\rm{j}}{\phi }_{A}^{\text{'}})\\ {B}^{\text{'}}={b}^{\text{'}}\exp ({\rm{j}}{\phi }_{B}^{\text{'}})\\ {C}^{\text{'}}={c}^{\text{'}}\exp ({\rm{j}}{\phi }_{C}^{\text{'}})\\ {D}^{\text{'}}={d}^{\text{'}}\exp ({\rm{j}}{\phi }_{D}^{\text{'}})\end{array}$$

After simplifying, the interference light intensity with the broad spectrum is given by

12 $$\begin{array}{rcl}{I}_{s} & = & {\int }_{-\infty }^{+\infty }\mathop{G}\limits^{\frown {}}(v){I}_{s}(v)dv\\  & = & {\int }_{-\infty }^{+\infty }\mathop{G}\limits^{\frown {}}(v)\{\begin{array}{l}1+2{\rm{\Delta }}{\theta }_{1}{\rm{\Delta }}{\theta }_{2}[a{\text{'}}^{2}(v)-b{\text{'}}^{2}(v)+c{\text{'}}^{2}(v)-d{\text{'}}^{2}(v)]\\ +(1+4{\rm{\Delta }}{\theta }_{1}{\rm{\Delta }}{\theta }_{2})a\text{'}(v)c\text{'}(v)\sin [{\phi }_{A\text{'}}(v)-{\phi }_{C\text{'}}(v)]\\ +(1-4{\rm{\Delta }}{\theta }_{1}{\rm{\Delta }}{\theta }_{2})b\text{'}(v)d\text{'}(v)\sin [{\phi }_{B\text{'}}(v)+{\phi }_{D\text{'}}(v)]\end{array}\}dv\end{array}$$

where $\hat{{G}}(\nu )$ is the normalized power spectral density (NPSD) of the light source. After adding SSF, the RME of D-IFOG is given by

13 $$RME={\rm{\Delta }}{\varphi }_{Bs}\cdot k$$

Where *k* is the scale factor. According to equation (9)

14 $${\rm{\Delta }}{{\varphi }}_{Bs}=\arcsin (\frac{{I}_{s}}{2}-1)$$

Introduce the suppression effect δ, which is the ratio of the RMEs of D-IFOG after and before adding the SSF

15 $$\delta =|\frac{{\rm{\Delta }}{\varphi }_{Bs}\cdot k}{{\rm{\Delta }}{\varphi }_{B}\cdot k}|\times 100 \% =|\frac{{\rm{\Delta }}{\varphi }_{Bs}}{{\rm{\Delta }}{\varphi }_{B}}|\times 100 \% $$

Where Δ*ϕ*_*B*_ is the phase difference of CW light and CCW light without SSF. As shown in the equation (15), for δ < 1, as δ decreases, the Δ*ϕ*_*Bs*_ becomes small too. It means a stronger suppression for the error. While in situation that δ > 1, it means the Δ*ϕ*_*Bs*_ is enlarged after adding a SSF.

For a better suppression effect, it is necessary to get the situation in which δ→0. As shown in equation (14), there must be *I*_*s*_→2. In equation (12), the integrity within the spectrum is substituted by the calculation of average wavelength, then the equation (12) can be further simplified as

16 $$\begin{array}{rcl}{I}_{s} & = & 1+2{\rm{\Delta }}{\theta }_{1}{\rm{\Delta }}{\theta }_{2}[a{\text{'}}^{2}(\bar{v})-b{\text{'}}^{2}(\bar{v})+c{\text{'}}^{2}(\bar{v})-d{\text{'}}^{2}(\bar{v})]\\  &  & +(1+4{\rm{\Delta }}{\theta }_{1}{\rm{\Delta }}{\theta }_{2})a\text{'}(\bar{v})c\text{'}(\bar{v})\sin [{\phi }_{A\text{'}}(\bar{v})-{\phi }_{C\text{'}}(\bar{v})]\\  &  & +(1-4{\rm{\Delta }}{\theta }_{1}{\rm{\Delta }}{\theta }_{2})b\text{'}(\bar{v})d\text{'}(\bar{v})\sin [{\phi }_{B\text{'}}(\bar{v})+{\phi }_{D\text{'}}(\bar{v})]\end{array}$$

In order to obtain the parameters of the SSF, we need to solve the equation bellow,

17 $$\begin{array}{c}2{\rm{\Delta }}{\theta }_{1}{\rm{\Delta }}{\theta }_{2}[a{\text{'}}^{2}(\bar{v})-b{\text{'}}^{2}(\bar{v})+c{\text{'}}^{2}(\bar{v})-d{\text{'}}^{2}(\bar{v})]\\ +(1+4{\rm{\Delta }}{\theta }_{1}{\rm{\Delta }}{\theta }_{2})a\text{'}(\bar{v})c\text{'}(\bar{v})\sin [{\phi }_{A\text{'}}(\bar{v})-{\phi }_{C\text{'}}(\bar{v})]\\ +(1-4{\rm{\Delta }}{\theta }_{1}{\rm{\Delta }}{\theta }_{2})b\text{'}(\bar{v})d\text{'}(\bar{v})\sin [{\phi }_{B\text{'}}(\bar{v})+{\phi }_{D\text{'}}(\bar{v})]=1\end{array}$$

Where Δ*θ*_1_ and Δ*θ*_2_ are constant. This is a very complicated transcendental equation. Every variable is the product of several matrixes in equation (2) and (6), and is closely related to the parameter Δ*β*_*s*_ and *φ*_*s*_, as well as the length of the i-th piece dz. So the solutions are not explicit and can be solved only through numerical calculations. The solution procedure and results will be given in section 3.

## Solution and simulation of the optical model for suppressing the RME

**Figure 3:**
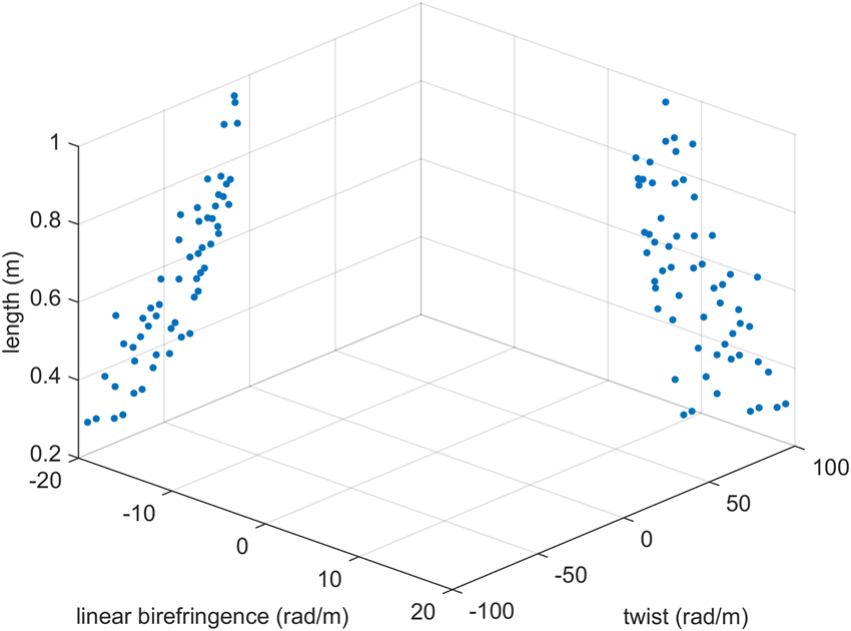
Spatial distribution of the solutions in the scanning range of parameters.

For one certain type of D-IFOG, we simulate and calculate the optical model for suppressing the RME. Firstly, set the length of the SSF to zero (namely there is no SSF), and set the orientation of the radial magnetic field arranging from 0° to 360°. Then the RME of the original D-IFOG is obtained. Secondly, set the range of the length, the linear birefringence, and the twist of the SSF, and scan these three parameters in scope respectively. If the maximal RME is zero or less than ten percent of the original D-IFOG (that is δ < 10%) with one set of the parameters, this set of parameters is one of the solutions satisfied with the suppression effect. Finally, the solution space is gotten as shown in Fig. 3 through scanning these parameters. The solution space contains all of the parameter combinations. They are not only satisfied with the suppression effect, but also constrained by the scanning range and scanning step of three parameters (length, linear birefringence, twist). As shown in Fig. 3, three axes represent length, linear birefringence and twist of the SSF.

**Figure 4:**
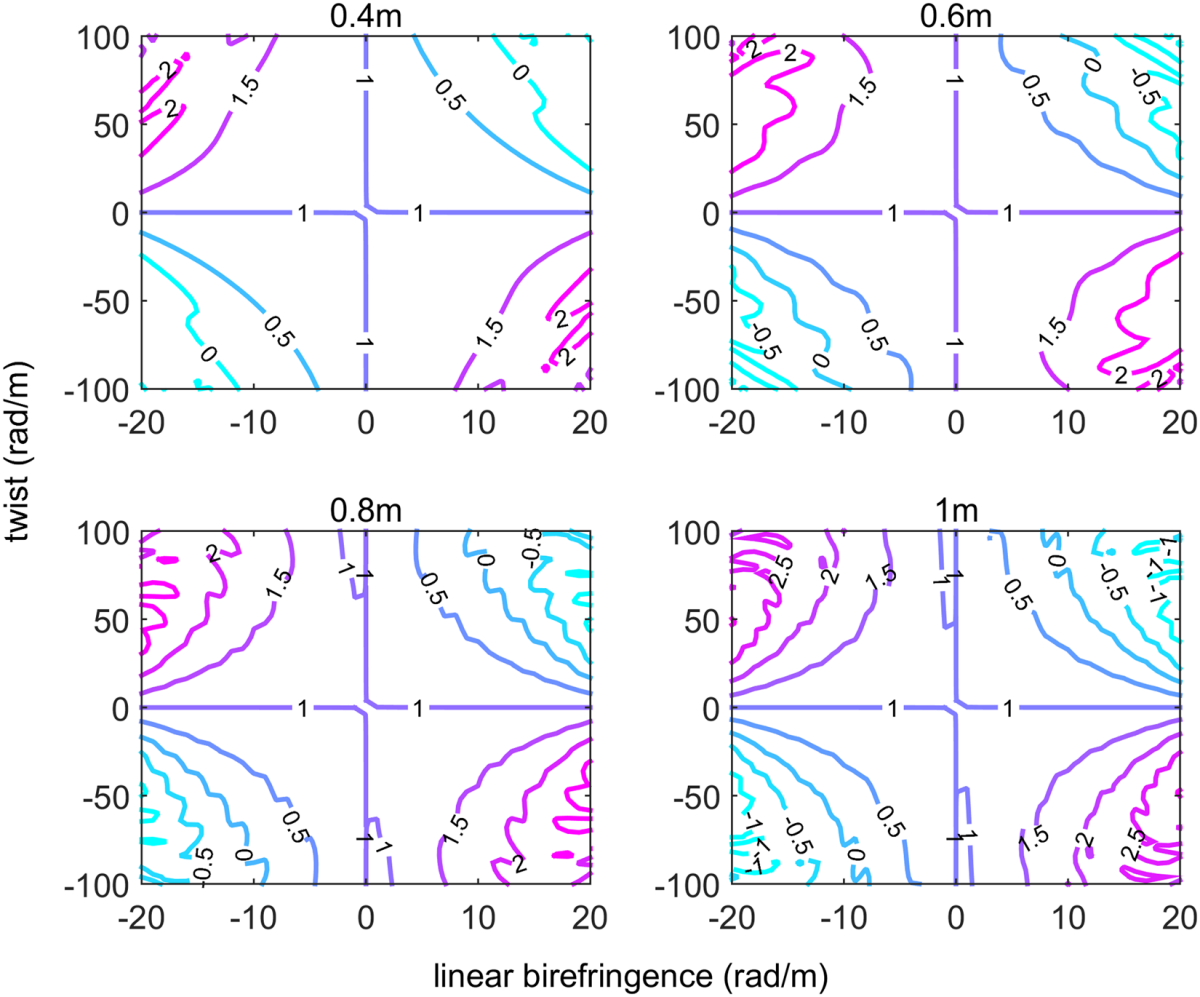
Relationship between the parameters of SSF and the RME of D-IFOG.

Figure 4 is the contour map of the RME after adding the SSF. It adopts several two-dimensional mappings to show the relationship between the parameters of the SSF and the RME of D-IFOG. The lengths of the SSF in four subgraphs are 0.4 m, 0.6 m, 0.8 m and 1.0 m respectively. In each subgraph, the horizontal axis and vertical axis represent linear birefringence and twist. The label value on the curve shows the RME after adopting the optical model and the unit is (°/h/Gs).

As shown in Fig. 4, the RME with SSF inside the system has obvious relationship with each parameter of SSF. Comparing the curves of zero RME in four subgraphs, we know that as the length of the SSF increases, the needed linear birefringence and twist become small. For the curve of zero RME in any subgraph, it is centrosymmetric about the origin. In addition, we can also see in Fig. 4 that, for a certain suppression result, a smaller (or larger) twist is needed if the linear birefringence becomes larger (or smaller). These conclusions will provide guideline for practical operation in experiment.

**Figure 5:**
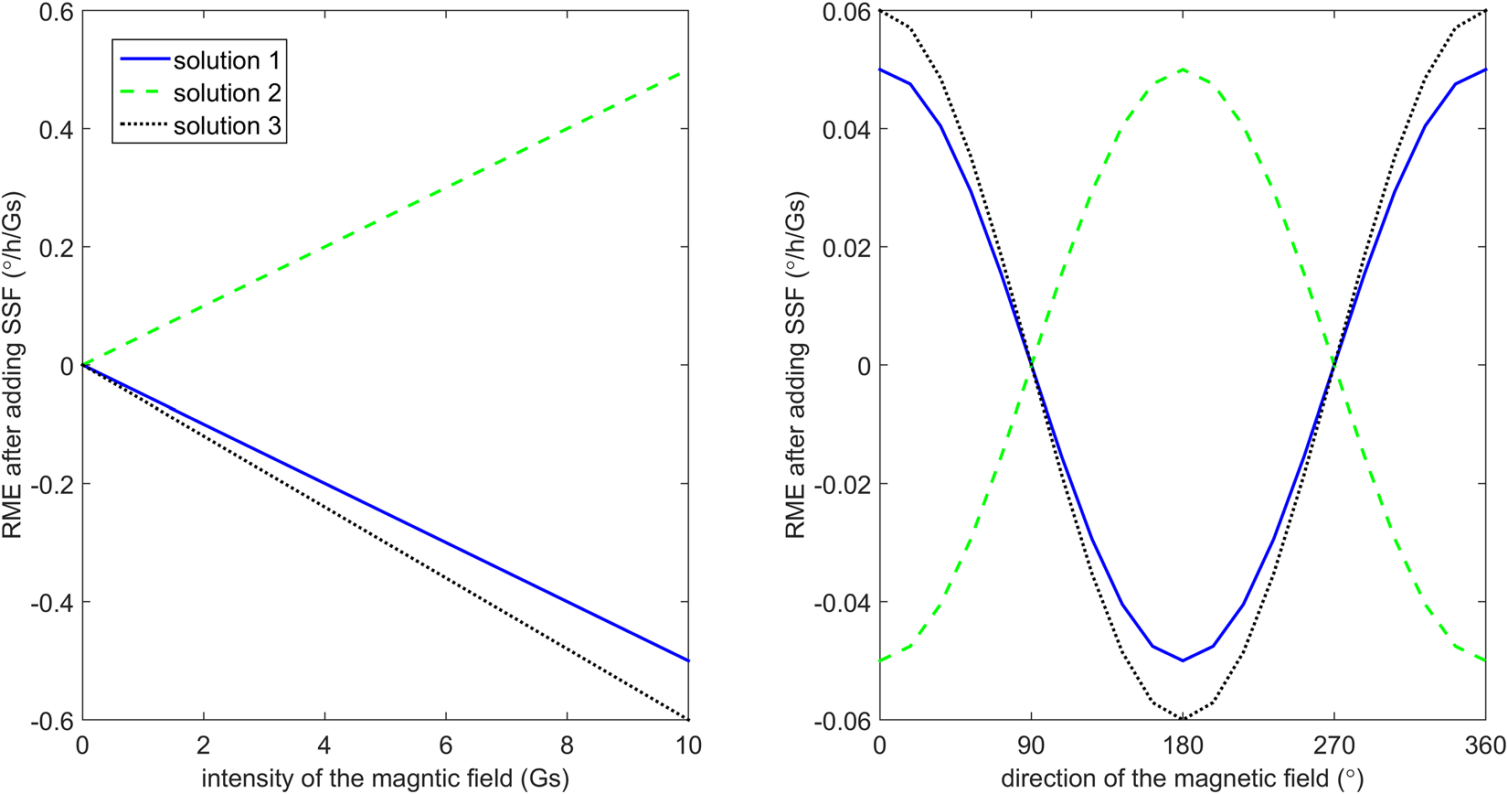
Suppression results after adding SSF.

The RME is about 1°/h/Gs for one certain type of D-IFOG. Three solutions of SSF are used to simulate the suppression results. The intensity of magnetic field varies from 1 to 10 G and the orientation of magnetic field changes from 0 to 360°. The results are shown in Fig. 5.

As shown in Fig. 5, for one solution (a set of parameters), after adding SSF, the RME is still linear with the intensity of the magnetic field and varies as a cosine function of the orientation of the magnetic field. That is, the SSF is equal to a zoom system and doesn’t change the orientation of the magnetic sensitive axis (the orientation of the maximum RME). The maximum RMEs for the three solutions are all no larger than 0.06°/h/Gs, which is satisfied with the requirement of the suppression effect.

## Experimental verification of the optical method

**Figure 6:**
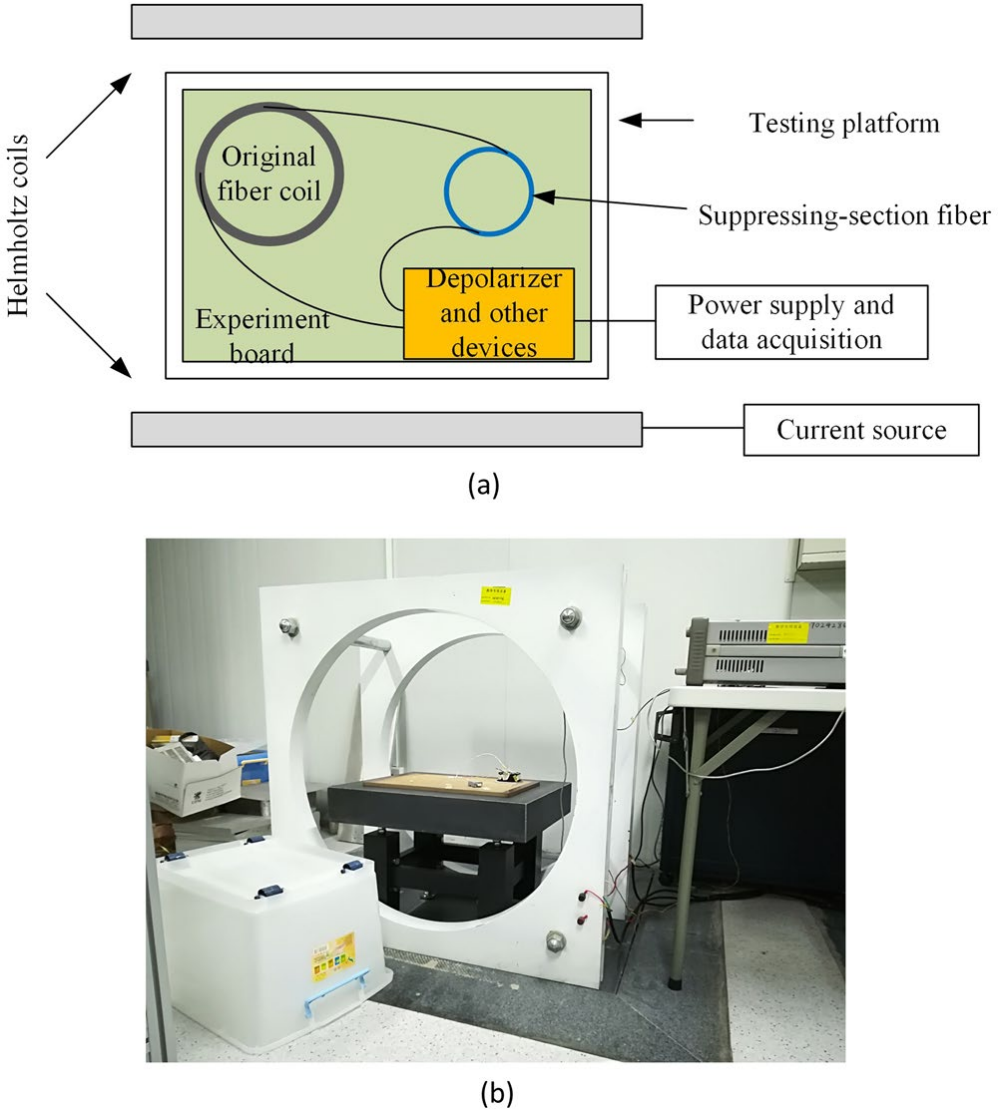
(**a**) Schematic diagram of the experimental facilities. (**b**)Photograph of the experimental system.

The schematic diagram of the experimental facilities is shown in Fig. 6(a) and the objects are shown in Fig. 6(b). The Helmholtz coils can produce a uniform magnetic field with Φ250 mm × 250 mm area in the center. These Helmholtz coils are powered by an adjustable current source and the intensity of the magnetic field can be varied from 0 to 20 G. Testing platform and Helmholtz coils are placed on the horizontal isolate foundation. D-IFOG, SSF and other devices are placed on the experiment board which is put on the testing platform. During experiments, the plastic box at the left bottom in the Fig. 6(b) is put over the experiment board to reduce noises caused by the air disturbance. The D-IFOG is powered at least 1 h before each experiment to avoid the influence of the starting drift. The experiment is divided into two steps. Firstly, the radial magnetic character of the original D-IFOG is tested, including the intensity and orientation of the maximum RME. Secondly, the SSF is added in the D-IFOG, and appropriate operation such as twisting and pressing is applied to this fiber. Then the radial magnetic character of the D-IFOG with SSF is gotten to analyze the suppression effect.

**Figure 7:**
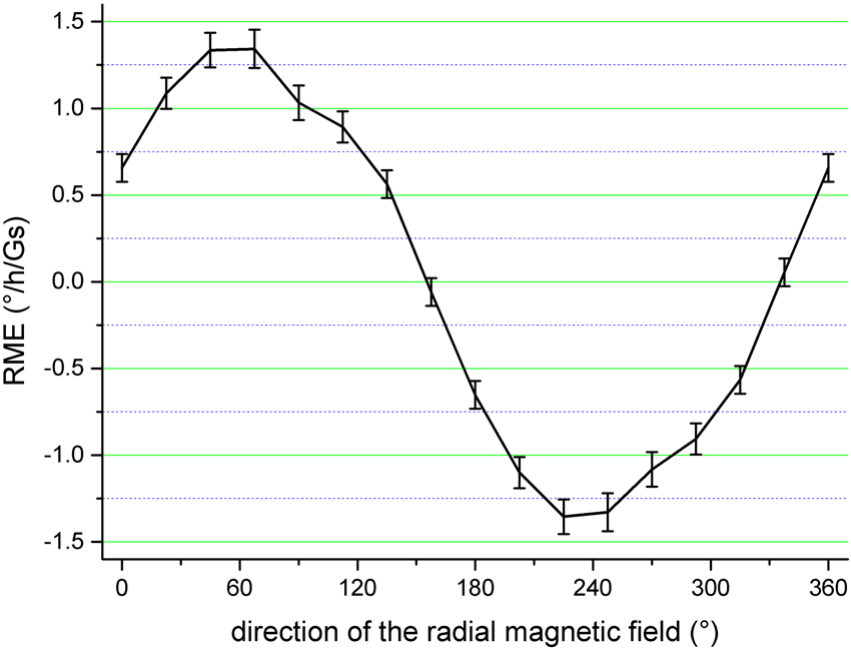
RME of the original D-IFOG with magnetic field in different orientations.

In step 1, the RME of the original D-IFOG is measured in radial magnetic fields with a same intensity but different orientations. Then the sensitive axis and the maximum value of the RME can be obtained. The orientation of the magnetic field is indicated by the scale line on the testing platform. There are eight scale lines representing sixteen orientations with an angle interval of 22.5°. The result of the experiment is shown in Fig. 7. The RME shows a proximately sinusoidal relation with the orientation of the magnetic field. We get a maximum RME of about 1.3°/h/Gs and a magnetic sensitive axis at 60° and 240°. In Fig. 7, the vertical line at each testing point represents the test error, which is twice of the standard deviation σ. So the test value can be expressed as the average with a deviation of ±σ. σ is about 0.1 °/h/Gs.

**Figure 8:**
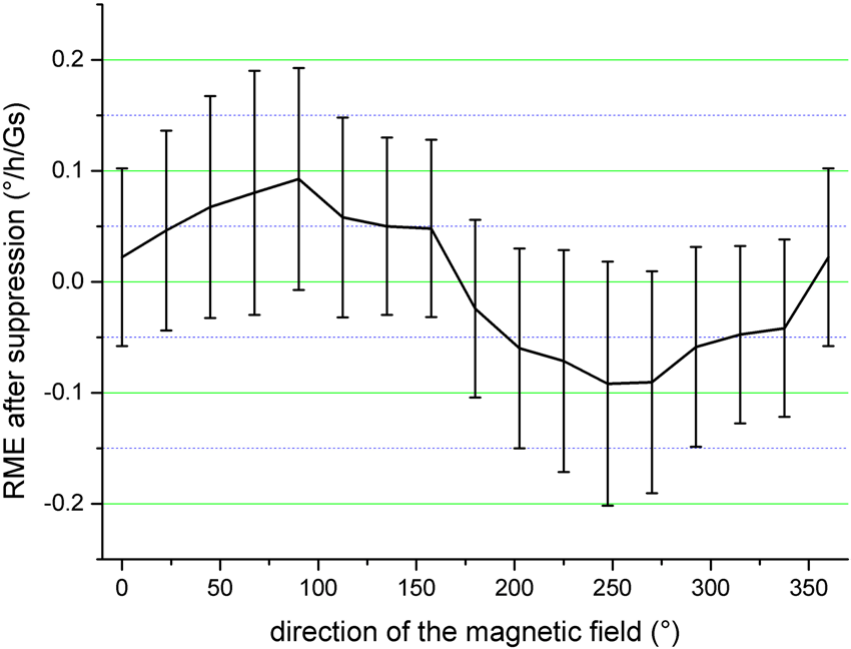
RME after adding the SSF.

Then the SSF is added in the D-IFOG and is looped and pressed properly. The SSF is fixed once it is adjusted to the appropriate position. Then the magnetic character is tested again. The result is shown in Fig. 8.

Comparing Fig. 7 wih Fig. 8, we get the suppression effect in experiment as

18 $$\delta =\frac{0.09}{1.35}\times 100 \% \approx 7 \% $$

This indicates that with proper parameters of SSF, a suppression effect less than 10% can be obtained. After suppression, the orientation of the magnetic sensitive axis remains the same. The RME still remains a sinusoidal relationship with the orientation of the magnetic field. The experimental results agree with the simulation in section 3.

## Conclusion

In this paper we put forward an optical method for suppressing RME in a D-IFOG. The RME is reduced by adding a SSF in the D-IFOG system. The theoretical model of the RME in D-IFOG with SSF is shown. By numerical calculations, the solutions for the parameters of the added SSF are obtained. Three solutions are chosen to simulate the suppression results. Then an experimental system is set up and the experimental results shows excellent agreement with the simulation. Experiments prove that, with this method, the RME can be reduced to one-tenth of the original. The work shows great possibility to improve the environmental adaptability for medium-to-high precision D-IFOG.

### Data availability

The datasets generated during the current study are available from the corresponding author on reasonable request.
